# Medical Schools in London, for 1846-7

**Published:** 1846-11

**Authors:** 


*Medical Schools in London, for* 1846–7.—To judge from the announcements which have been made through our advertising columns for the last three weeks, it would appear that the postponement of the medical reform measures has had very little influence on the Schools. The number of these, although more than sufficient for the wants of the profession, is nearly as great as at any previous period; and if a few "professors" have disappeared from the scenes of their former labors, their "chairs" have been filled with a facility and rapidity which would lead to the supposition that the outcry respecting the ill-paid services of medical teachers, is quite unfounded. Prizes in the shape of gold, silver, and bronze medals, testimonials of honour, and scholarships, are announced among the good things to which the student has to look forward as the reward of industry : and some of the metropolitan and provincial schools vie with each other in the rich promises thus held out to the youthful aspirants for medical honours. In our judgment this system is carried too far: these prizes, by the profusion with which they are bestowed, are too frequently made to serve as a meretricious bait to attract students to particular institutions. If the benefits to be derived from instruction at a school, were always proportioned to the number and value of medals, &c., distributed, there might be some reason to defend the system ; but it is well known that they too often serve as objects to allure persons to schools which are entirely deficient in the means of practical instruction. We have heard of one establishment where the prizes were as numerous as the students; in fact, there were no blanks ! This system leads to a degrading kind of competition among medical schools, and has the injurious effect of creating a special class of students under the name of prizemen, who work rather to obtain these glittering baubles, than to acquire a sound knowledge of their profession.

The reform of the medical profession must begin with a sweeping reform in the present system of medical tuition. Apprenticeships, whereby under an act of Parliament students were compelled to waste some of those years which were best adapted for preliminary education and the acquisition of elementary professional knowledge, are now all but abolished. One medical school has this year adopted the novel practice of including classics, mathematics, indentures of apprenticeship, &c., in a general fee ; and indeed the exact sum required to convert a man into a surgeon and apothecary, is now made as much a matter of certainty as the purchase of a government annuity ! Apprenticeships have not been for a long time practically enforced. They are nominally entered into, but by a mutual arrangement the student commonly finds the means of including the three years' term of medical education, in the period assigned to the apprenticeship.

A student is thus thrown young upon the stage of professional study, and unless he have a parent or relative in the profession, the choice of a school is commonly left to himself. Much of his future success depends upon the selection which he may make. He may meet with bad teachers and bad associates—he may enter himself to an "establishment" such as the defunct Sydenham College, which in its day, judging by the exterior, consisted of two coffee-shops thrown into one ; yet here was an attractive name—a "College"—the "chairs" were filled by "professors" in the respective branches of medical science, the fees were usually low ; and there was a flood of prizes announced for distribution at the termination of the medical session ! It is creditable, however, to the good sense of the class of medical students, that allurements of this kind generally fail to procure that support which is essentially necessary to the maintenance of such establishments.

A *medical* school without a hospital or infirmary attached to it, is necessarily deficient in the means of practical instruction ; and under a proper system of medical education, this form of theoretical instruction, however good it may be in some rare cases, should, in our opinion, be entirely abolished. Some branches of medical science may, it is true, be taught in establishments unconnected with hospitals ; but the practice has the evil effect of compelling a student to follow two sets of teachers, and entirely neglect either his lectures or the wards of the hospital. We are satisfied, from a longe acquaintance with students, that there can be no worse plan than that of attending lectures at one institution and hospital practice at another; hence, in the selection of a school, they should especially observe whether it be or be not provided with a hospital or infirmary capable of furnishing efficient practical instruction. They may be well assured that no excellence in teachers, apart from a ready access to the wards of a hospital, will recompense them for the deficiency. Upon the choice of a hospital it is unnecessary for us to make any remark. Among the many excellent institutions of this kind which now exist in London, it matters little which a student may select ; for there is no hospital school which is not amply provided with the means of giving sound practical instruction.

On one point, however, we wish to offer a word of caution. Medical education has of late years unfortunately assumed the character of commercial speculation. Competition has been carried to that degree, that in some instances it is quite certain, from the lowness of the fees charged for attendance, there can be no remuneration for the proper performance of the duties. The courses prescribed by the Colleges and Halls are guaranteed at a certain low sum in one payment ; but it is clear that, unless there be a very large number of students in the school, there will be a great temptation to laxity and neglect on the part of the teachers ; and although a low consolidation fee for a three years' course of study may operate as a great inducement to enter to a particular school, yet the student may find in the end that he is paying only for printed certificates, and that if he really require to be instructed in his profession, it will be necessary for him to go elsewhere. True economy will therefore be found in selecting a good and efficient school in the first instance.—*Ibid.*

